# Thalamocortical white matter microstructure and cognition following mild traumatic brain injury: A fixel-based analysis

**DOI:** 10.1007/s00429-026-03193-7

**Published:** 2026-09-26

**Authors:** Maggie Baird, Marc L. Seal, Richard Beare, Joseph Yuan-Mou Yang, Jacqueline F. I. Anderson

**Affiliations:** 1 Melbourne School of Psychological Sciences, The University of Melbourne, Redmond Barry Building, Melburne, VIC 3052 Australia; 2 Developmental Imaging, Clinical Sciences, Murdoch Children’s Research Institute, Parkville, Melbourne, Australia; 3 National Centre for Healthy Ageing and Peninsula Clinical School, Monash University, Melbourne, Australia; 4 Department of Neurosurgery, Neuroscience Advanced Clinical Imaging Service (NACIS), The Royal Children’s Hospital, Melbourne, Australia; 5 Neuroscience Research, Murdoch Children’s Research Institute, Parkville, Melbourne, Australia; 6 Department of Pediatrics, The University of Melbourne, Parkville, Melbourne, Australia; 7 Psychology Department, The Alfred hospital, Prahran, VIC Australia

**Keywords:** Mild traumatic brain injury, Thalamus, Diffusion weighted imaging, Fixel based analysis, Fibre density, Cognition, Neuropsychology

## Abstract

The thalamus, an essential hub for cognitive integration across distributed brain networks, has emerged as a key structure implicated in mild traumatic brain injury (mTBI). However, while white matter microstructural injury is considered a core pathophysiological substrate of mTBI, few studies have examined thalamocortical microstructure following injury. The present study applied an advanced diffusion MRI-based white matter microstructural model, fixel-based analysis (FBA), to examine thalamocortical white matter microstructure and its associations with cognitive performance at approximately two-months following mTBI. The final analysed dataset included 39 mTBI patients and 28 trauma control (TC) patients aged 18–60 years, who were recruited following hospital admission for physical injury. Participants completed a range of cognitive tests and an MRI protocol including diffusion weighted sequences at 6–12 weeks following injury. Thalamocortical tracts were delineated bilaterally using the TractSeg algorithm, an automated white matter bundle segmentation tool. While whole-brain FBA showed no statistically significant differences in fixel metrics between groups, tract-based analyses showed that mTBI was associated with reduced fibre density (FD) of the right thalamo-precentral (BF_10_ = 6.849), right thalamo-parietal (BF_10_ = 3.708) and left thalamo-precentral (BF_10_ = 1.276) tracts. Despite these structural differences, the groups were comparable with respect to performance on cognitive tests. However, models with interaction effects indicated that associations between thalamocortical FD and cognitive performance differed between groups, most frequently on measures of processing speed and attention. Together, these findings indicate at this stage of recovery, changes in white matter microstructure are detectable in thalamocortical tracts, and that structure-cognition relationships may differ between mTBI and TC groups even in the absence of objective cognitive impairment.

**Supplementary Information:**

The online version contains supplementary material available at https://doi.org/10.1007/s00429-026-03193-7.

## Introduction

Diffusion-weighted imaging (DWI) has been instrumental in detecting tissue alterations as a consequence of mild traumatic brain injury (mTBI), which has led to the view that white matter microstructural injury is a core pathophysiology feature (Lindsey et al. 2023). At the time of impact, inertial loading imparts energy to the head and causes the brain to rapidly shift in the skull, causing stretching injuries throughout white matter networks (Dashnaw et al. 2012; Greve and Zink 2009). Secondary neurometabolic and neurochemical processes can further exacerbate the diffuse microscopic axonal injury through excitotoxicity, ionic imbalance and impaired mitochondrial function (Giza and Hovda 2014). The resultant white matter abnormalities have been shown to be associated with the cognitive dysfunction that occurs following injury (Oehr and Anderson 2017). For most individuals, objective cognitive impairments are transient and demonstrate progressive resolution within the first weeks following injury, such that overt cognitive deficits are not expected by three-months following injury (Karr et al. 2014). However, while group-level cognitive recovery occurs by this time point, neuroimaging studies have shown that white matter microstructure abnormalities may persist beyond this period, which has motivated interest in the associations between structure and cognition toward the end of the typical recovery period.

Structural lesions detectable using the neuroimaging methods routinely applied in clinical settings are uncommon following mTBI (Bigler et al., 2016). However, the advent of DWI, and its associated modelling technique diffusion tensor imaging (DTI), has enabled the characterisation of subtle white matter abnormalities. A substantial body of literature has reported heterogenous changes in DTI-derived metrics in mTBI, which, while suggestive of white matter microstructural abnormalities, have not been consistent with respect to the nature of these changes (Lindsey et al. 2023). This may relate to methodological limitations of the DTI model, which is limited in its capacity to resolve complex crossing fibres and conflates multiple biological processes into single derived metrics (Farquharson et al. 2013). Given that crossing fibres are highly prevalent in the brain, these limitations significantly constrain the interpretability of subtle postinjury white matter alterations (Jeurissen et al. 2019).

Fixel-based analysis (FBA) is an advanced diffusion MRI framework which addresses these limitations by enabling the quantification of distinct fibre populations within voxels, called “fixels”, allowing the assessment of white matter properties at the subvoxel level (Dhollander et al. 2021). FBA yields fibre-specific metrics including fibre density (FD), fibre cross-section (FC), and a combined measure of both (FDC), which are highly sensitive to white matter morphology. The FBA method therefore offers significant advantages over DTI, which averages diffusion properties across all fibre populations within a voxel, including greater specificity in regions containing complex crossing fibres and increased biological sensitivity to fibre-specific properties (Raffelt et al. 2017). Despite these critical methodological improvements, applications of FBA within the mTBI population have been limited. Existing studies have reported increased FD and FC in acute mTBI (≤ 12 days postinjury), no group differences in any metrics at 7-months postinjury, and reduced FD and FDC at 10-months postinjury within specific cortical tracts (Burnett et al. 2025; Mito et al. 2022; Wallace et al. 2020). Therefore, FBA appears to have utility in assessing white matter microstructural abnormality following mTBI. Collectively, these findings also suggest that the direction of microstructural change may vary across the recovery trajectory, although further investigation is required.

The thalamus, through its dense reciprocal structural connectivity with the cortex and other subcortical regions, occupies a central hub-like position within large-scale brain networks that enables it to actively shape brain-wide dynamics and support cognition (Hwang et al., 2021; Shine et al., 2023; Biesbroek et al., 2024; Dehghani and Wimmer, 2019). The importance of these thalamocortical connections is further supported by DWI studies demonstrating that microstructural abnormalities within thalamocortical white matter are associated with cognitive impairment in clinical populations including multiple sclerosis and dementia (Bernabéu-Sanz et al., 2021; Delli Pizzi et al., 2015). The thalamus’s central, midline anatomical position and integration within long-range white matter tracts may also confer particular vulnerability to the rotational forces of mTBI, consistent with reports of altered thalamic volume, structural and functional connectivity, and white matter microstructure following injury (Aoki and Inokuchi 2016; Baird et al., 2024; Bigler, 2021; Woodrow et al., 2023). However, whether microstructural alterations occur within thalamocortical white matter following mTBI, and how these relate to cognitive outcome, remains unknown. As a fibre-specific technique, FBA may provide novel insights into thalamocortical white matter microstructure and its relationship with cognition following mTBI.

The present study had two primary aims: First, to characterise features of thalamocortical white matter in an mTBI group compared to a trauma control group using the FBA method, in order to provide a sensitive and fibre-specific assessment of microstructure. Second, to examine associations between thalamocortical white matter microstructure and performance on cognitive tests following injury. These aspects of outcome were studied at approximately two-months following mTBI, a time point expected to be toward the end of the cognitive recovery period.

## Materials and methods

### Participants

Two groups of participants, mTBI (*n* = 42) and trauma controls (TCs; *n* = 29) were recruited after being admitted to either the Royal Melbourne Hospital or The Alfred hospital in Melbourne, Australia due to trauma-related physical injury. Participants were recruited between 2017 and 2023. Participants were classified into the mTBI group if they sustained a traumatic physical injury and also fulfilled the World Health Organisation criteria for definition of mTBI: Glasgow Coma Scale score between 13 and 15 at 30 min post-injury, and one or more of the following symptoms: < 24 h post-traumatic amnesia; < 30 min loss of consciousness, impaired mental state at time of accidence (e.g., confusion, disorientation); and/or transient neurological deficit (e.g., seizure). The presence of mTBI was diagnosed by trained recruiters and hospital records were not relied upon given variation in terminology and application of criteria. Participants were included in the TC group if they sustained a traumatic physical injury with no head strike. All participants fulfilled the following inclusion criteria: ages 18–60, no neurological history, no active psychiatric episode at time of injury, no psychiatric treatment and/or symptoms for the preceding 12 months, no current or historical heavy alcohol use (average > 6 standard drinks/day), no current or historical intravenous drug use, and conversational English fluency. Injuries that occurred in the context of assault and self-harm were excluded. Veterans and professional athletes were excluded. Additionally, mTBI participants were excluded if they had previously sustained more than two mTBIs or been admitted to hospital for a prior mTBI, or ever sustained a traumatic brain injury of moderate-severe severity. TC participants were excluded if they had ever sustained a traumatic brain injury of any severity. All participants provided informed consent. The project was approved by The Alfred and The Royal Melbourne Hospital Human Research Ethics Committees.

### Measures

#### Clinical measures

A series of cognitive measures were completed by all participants. Raw scores for each measure were centred and scaled to aid in interpretation of statistical parameters.

#### Digit span

The Digit Span subtest of the Wechsler Adult Intelligence Scale- Fourth Edition (Wechsler 2008) was used to provide measures of basic and higher order attention (working memory). The digits forward (DSF) component, which provides a measure of basic attention, requires the individual to repeat digits of increasing length. The digits backward (DSB) component, which provides a measure of higher order attention, requires the individual to repeat digits of increasing length in the opposite order they are presented.

#### Symbol digit modalities test (SDMT)

The SDMT (Smith 1973) is a measure of processing speed. Individuals are provided a key with nine symbols corresponding to numbers and are required to transcribe a series of symbols as quickly as possible. The total score comprised the number of correctly transcribed numbers completed within the 120 s time limit.

#### Rey auditory verbal learning test (RAVLT)

The RAVLT (Lezak 2004) was used a measure of new verbal learning and memory recall. Individuals are verbally presented with a 15-item word list A over five learning trials. Following presentation of a 15-item distractor list B and a 20-minute delay, individuals are required to freely recall words from list A (Trial 7). Scores obtained included the total learning score (summed total number of correctly recalled words over Trials 1–5), and the number of words recalled after a 20-minute delay (Trial 7).

#### Victoria stroop test

The Stroop Test (Regard 1981) involves a speeded naming task with three components. Respondents are required to name the ink colour of dots (Dots trial), ink colour of neutral words (Words trial), and ink colour of incongruent colour names (Colour-Words trial). An interference score was calculated as a measure of cognitive control (executive function) by dividing the number of seconds to complete the Colour-Words trial by the number of seconds to complete the Dots trial.

#### Trail making test (TMT)

The TMT (Sánchez-Cubillo et al. 2009) is a two-component measure of attention and set-shifting. Part A requires the individual to draw a line sequentially connecting a random array of 25 digits. Part B requires alternation between numbers and letters. Participants are instructed to complete each component as quickly as possible. The TMT ratio score (B/A) was chosen to calculate as it provides a measure of set-shifting (executive function) that is independent of psychomotor speed, and has been shown to better isolate this function compared to the B-A score (Arbuthnott and Frank 2000).

### MRI data acquisition

MRI data was acquired on a 3T Siemens Magnetom Prisma 3 Tesla MRI machine (PRISMA Siemens, Erlangen, German) using a 64-channel head coil at the Baker Heart and Diabetes Institute, Melbourne, Australia. Structural T1-weighted images were acquired using the magnetisation prepared rapid gradient-echo (MPRAGE) sequence (240 × 256 mm acquisition matrix; FOV = 256 mm; 176 contiguous slices; 1mm^3^ isotropic voxel size; TR/TE = 2300/2.96 ms).

DWI data were acquired in the anterior-posterior phase encoded direction, with multiband accelerated EPI sequences for multishell acquisition (128 × 128 mm acquisition matrix; FOV = 256 mm; 75 contiguous slices; 64 b = 1000 s/mm^2^, 64 b = 3000 s/mm^2^, 4 b = 0 s/mm^2^; 2mm^3^ isotropic resolution; TR/TE = 4800/88 ms; MB factor = 3). A radiologist screened all scans at acquisition for abnormalities.

### MRI preprocessing

**Fig. 1:**
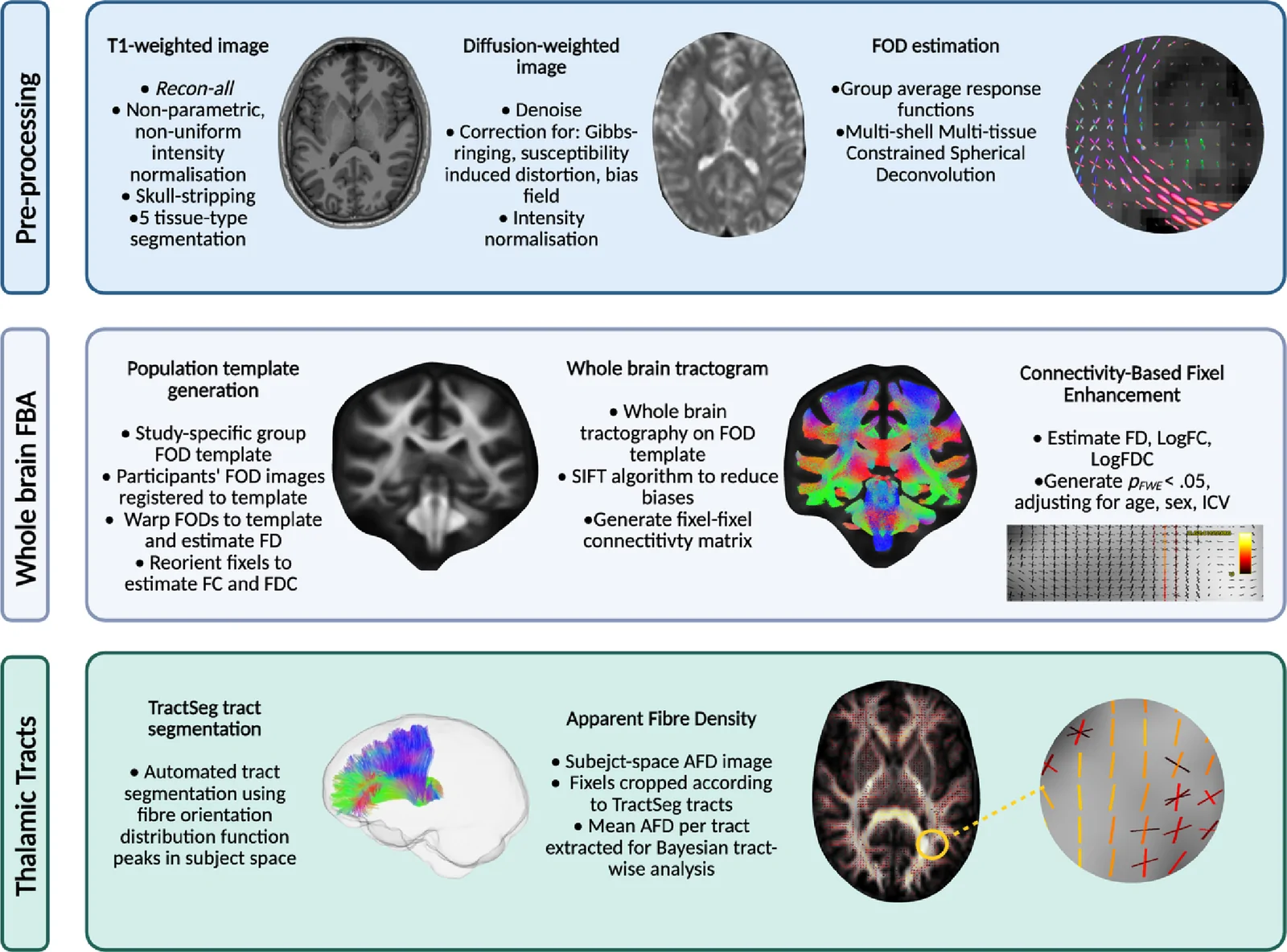
Overview of processing pipeline. *Note.* Following preprocessing, diffusion-weighted images were visually inspected for gross artifacts or preprocessing failures, and T1 images underwent quality control according to the method suggested by Klapwijk et al. (2019)

Briefly, each participant’s T1w images were reoriented to match the orientation of MINI152 and cropped using FSL functions (FMRIB’s Software Library; https://fsl.fmrib.ox.ac.uk/fsl). T1w images were processed using the standard *recon-all* processing pipeline in the FreeSurfer analysis suite release 7.4.1 (Fischl 2012), and estimated intracranial volume was derived. Multishell DWI data were pre-processed using a combination of the MRtrix3 software package (version 3.0.4; Brain Research Institute, Melbourne, Australia, www.mrtrix.org) and FSL (FMRIB’s Software Library; https://fsl.fmrib.ox.ac.uk/fsl). For each scan, these steps were: denoising, motion correction, correction for Gibbs ringing, correction for susceptibility-induced distortions using Synb0-DisCo (Schilling et al. 2019), bias field correction using the N4 algorithm (Tustison et al., 2010), and intensity value normalisation using *dwinormalise* (Tournier et al. 2019). Figure 1 presents an overview of the processing pipeline.

### Whole Brain Fixel-Based Analysis

FBA was performed using the recommended pipeline documented by MRtrix3 (v 0.3.0.4) to derive fixel metrics (Dhollander et al. 2021). Briefly, data were upsampled to an isotropic voxel size of 1.25 mm using cubic interpolation, and multi-shell multi-tissue constrained spherical deconvolution fibre-orientation distributions (FODs) were estimated at the voxel-level. Joint bias field and intensity normalisation were then performed (Jeurissen et al. 2014). A study-specific group-average template was computed using all participants and all participants’ FOD images were registered to the unbiased template. A whole-brain tractogram was generated from the FOD template (angle 22.5, maximum length 250, minimum length 10, power 1, 20 × 10^6^ streamlines, FOD amplitude cutoff 0.06), and the Spherical-deconvolution Informed Filtering of Tractograms (SIFT) algorithm was applied to reduce tractography biases in the whole-brain tractogram (2 × 10^6^ streamlines; (Smith et al. 2013).

### Tract of interest analysis

**Fig. 2:**
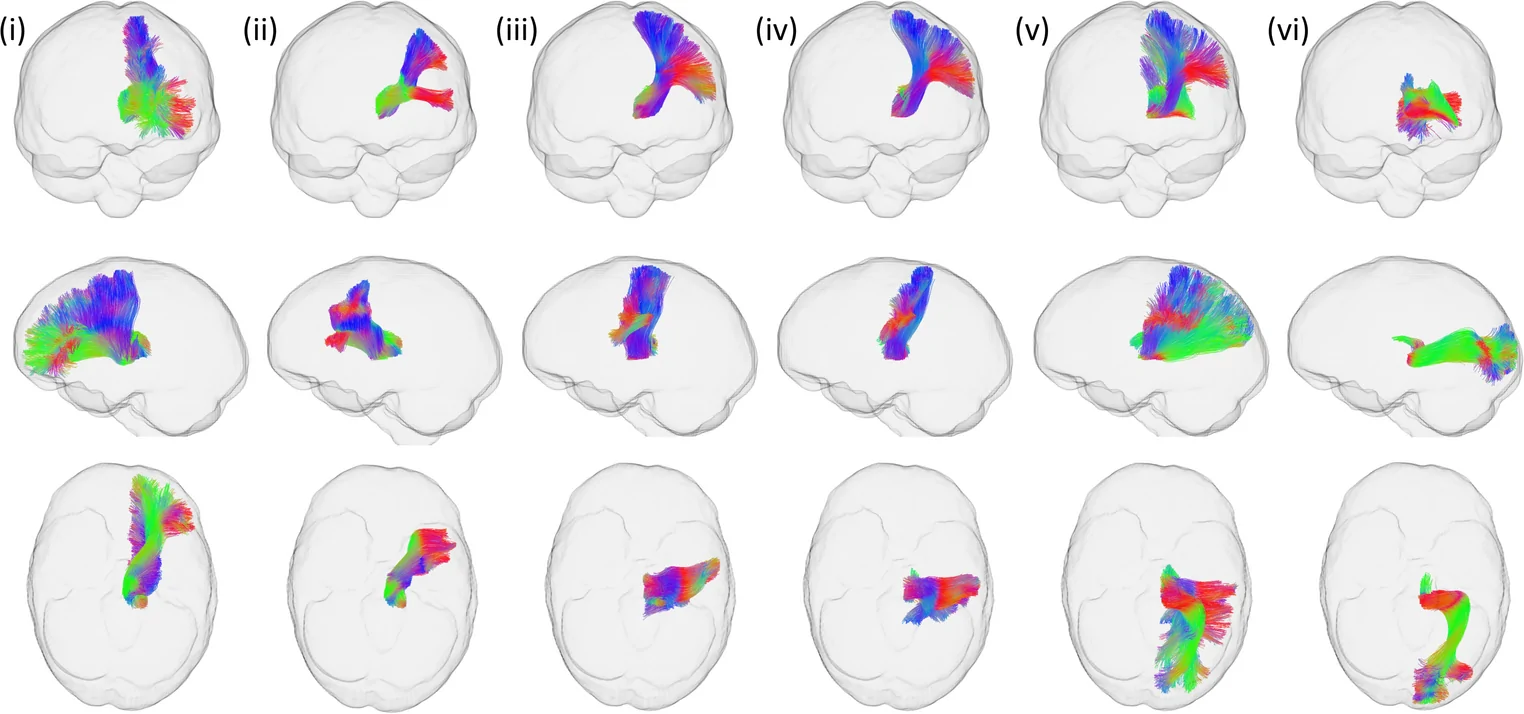
Thalamocortical tracts as segmented by TractSeg. Left hemispheric view of fibre pathways presented on coronal (top row), sagittal (middle row), and axial (bottom row) sides of a 3D brain reconstruction. Tracts of interest are presented as follows: (i) Thalamo-prefrontal, (ii) Thalamo-premotor, (iii) Thalamo-precentral, (iv) Thalamo-postcentral, (v) Thalamo-parietal, and (vi) Thalamo-occipital

The primary aim of this study was to investigate microstructural properties of thalamic tracts. To this end, we undertook tract of interest analyses by applying a widely validated tract segmentation method, *TractSeg*, which uses a convolutional neural network approach to delineate white matter tracts directly in the field of FOD functions (Wasserthal et al. 2018). Automated white matter tract segmentation was performed in subject-space using multishell constrained spherical deconvolution. The following bundle segmentations were delineated bilaterally for left and right hemispheres: Thalamo-prefrontal (T-PREF), thalamo-premotor (T-PREM), thalamo-precentral (T-PREC), thalamo-postcentral (T-POST), thalamo-parietal (T-PAR), and thalamo-occipital (T-OCC). To extract mean fibre density (FD) for each thalamic tract in native space, the Apparent Fibre Density (AFD) per fixel image was generated from the white matter FODs derived in the whole brain analysis. The AFD image represents the integral of each FOD lobe and therefore provides the diffusion-MRI estimate of intra-axonal volume for each fibre population (i.e., FD). All relevant tracts were reviewed for each participant and found to be successfully isolated. Figure 2 presents the segmented thalamic tracts for one mTBI individual.

### Statistical analysis

Whole brain fixel-based statistical analyses were computed at fixel-level across the whole-brain white matter (Raffelt et al., 2017). At each fixel, FD, log transformed FC and log transformed FDC were compared using a general linear model between mTBI and TC groups, adjusting for age, sex, and intracranial volume. Significant differences in fixel metrics were identified using connectivity-based fixel enhancement (CFE), and non-parametric permutation-based inference testing to compute family-wise error (FWE) corrected p-values. Fixels surviving FWE correction were visualised using the *mrview* tool in MRtrix3. Fixels were defined as statistically significant at *p*_*fwe*_ < 0.05.

A Bayesian framework was adopted for all remaining statistical analyses. Bayesian analysis tests relative evidence for a specified model, given the available data, and therefore quantifies the level of evidence in support of the absence of an effect against the alternative hypothesis model (i.e., the presence of an effect). The more commonly employed frequentist approach to statistical inference provides a limited measure of the strength of evidence against the null hypothesis and suffers from low power when applied to small sample sizes (Makowski et al. 2019). All Bayesian statistical analyses were conducted in JASP (version 0.19.3). As per guidelines for the reporting of Bayesian analyses (Makowski et al. 2019), for all statistical analyses we report: the median or mean of the posterior distribution and its associated credible interval (95% CI), which represents the point estimate for each parameter and the uncertainly around these estimates, and the associated Bayes Factors (BF_10_), which in this case are ratios of the alternative over the null hypotheses. For analyses consistent with recommendations, BF_10_ values less than 1/3 were considered as providing evidence in support of the null model; BF_10_ values between 1/3 and 1 were interpreted as offering inconclusive support for or against the null hypothesis; BF_10_ values between 1 and 3 were interpreted as providing robust but anecdotal evidence in favour of the alternative model, and values greater than 3 were interpreted as providing moderate and increasing evidence in favour of the alternative hypothesis (Makowski et al. 2019).

#### Covariates

To determine the inclusion of covariates in the main analyses, Bayesian correlations were performed to evaluate the potential association between tract-FD and age, tract-FD and time since injury, age and cognitive performance, and time since injury and cognitive performance. Bayesian independent samples t-tests were performed to evaluate the association between sex and tract-FD, and sex and cognitive performance.

#### Thalamocortical tract-based analysis

Group-wise differences in FD values for each tract were compared using Bayesian linear models, and the BF_10_ for the alternative hypothesis of a group difference in tract-FD was computed. Main tract-based analyses included sex and age as covariates. To examine whether thalamocortical tract-FD could predict performance on cognitive testing, Bayesian linear models were fitted, and the Bayesian inclusion factors for the addition of an interaction between group and tract-FD were computed. Given that we may have been underpowered to detect an interaction effect, relationships between tract-FD and cognitive performance were subsequently fitted for the mTBI and TC groups separately. For all main analyses, priors over parameters were set as normal, and model priors were uniform. For all main analyses, the null model included the relevant covariates, and the alternative model contained these predictors plus the inclusion term of interest (e.g., tract-FD for cognitive performance models).

## Results

Imaging data were obtained for 71 participants and successfully analysed in 67: One mTBI participant was excluded due to the incidental identification of a brain tumour. One TC and two mTBI participants were excluded due to an error in the diffusion sequence acquisition parameters and subsequent failure of FBA. The final analysed dataset included 67 participants (39 mTBI and 28 TC) who completed the protocol at 6–12 weeks following injury (range: 40–80 days, mean 56, sd 11). As has been reported previously, there were no differences between the mTBI and TC groups on demographic variables, injury-related variables, mood or post-concussion symptoms, or performance on cognitive tests (Baird et al. 2026). Table 1 presents demographic and injury-related variables.

**Table 1:** Demographic and injury-related variables for mTBI and TC groups

	mTBI (*n* = 39) M (SD)	TC (*n* = 28) M (SD)	Posterior Median [95% CI]	BF_10_
Demographic variables				
Age	37.2 (15.0)	37.5 (12.8)	– 0.21 [– 6.41, 6.11]	0.252
Gender (% F)	23	10		0.972
Education	13.0 (2.3)	13.3 (2.9)	– 0.24 [– 1.39, 0.87]	0.274
Injury-related variables				
Accident type %				0.998
MVA	15	24		
MBA	18	28		
Cycling	33	21		
Fall	13	17		
Other	21	10		
GCS %				
13	5			
14	28			
15	67			
PTA %				
< 5 min	46			
5–6 h	31			
6–24 h	23			
LOC %				
Not endorsed	23			
< 5 min	56			
> 5 min	21			
Time since injury (days)	57.9 (9.7)	56.1 (13.1)	1.57 [-2.50, 6.89]	0.305
Cognitive variables				
DSF	10.2 (2.6)	10.2 (2.9)	0.02 [-0.43, 0.47]	0.258
DSB	8.4 (2.2)	8.9 (2.4)	– 0.16 [– 0.62, 0.29]	0.349
RAVLT total	52.4 (9.4)	51.6 (11.0)	– 0.02 [– 0.47, 0.43]	0.264
RAVTL A7	11.5 (2.9)	10.4 (3.2)	– 0.25 [– 0.73, 0.20]	0.502
SDMT	66.3 (12.3)	65.3 (14.9)	– 0.01 [– 0.46, 0.44]	0.258
TMT ratio	2.7 (0.9)	2.7 (1.0)	– 0.07 [– 0.52, 0.38]	0.264
Stroop interference	1.9 (0.6)	1.7 (0.4)	– 0.34 [– 0.82, 0.11]	0.613

95% CI, 95% credible interval

*CCAMCHI* Cognitive Complaint After Mild Closed Head Injury, *DSB* Digit Span Backward, *DSF* Digit Span Forward, *F* female, *GCS* Glasgow Coma Scale, *IDP* Increasing Distractors Paradigm, *LOC* loss of consciousness, *M* mean, *MBA* motor bike accident, *mTBI* mild traumatic brain injury, *MVA* motor vehicle accident, *PTA* posttraumatic amnesia, *RAVLT* Rey Auditory Verbal Learning Test, *SD* standard deviation, *SDMT* Symbol Digit Modalities Test, *TC* trauma control, *TMT* Trail Making Test

### Covariate inclusion

The results of all covariate analyses are presented in the supplementary material. Age and sex were included in all main analyses given there was evidence that both variables were associated with tract-FD and cognitive performance, as is presented in Table 2. Time since injury was not included in the main analyses as it was not associated with tract-FD or cognitive performance, and there was no evidence of a difference between the two groups.

**Table 2:** Relationships between age and sex with tract-FD and cognitive performance

		Bayesian correlation (age)		Bayesian test of association (sex)		
		*r*	BF_10_	Male (m, sd)	Female (m, sd)	BF_10_
Tract	Hem					
T-PREF	R	– 0.40	35.21	0.354	0.373	2.070
	L	– 0.405	194.61	0.356	0.382	9.199
T-PREM	R	– 0.29	2.246	0.361	0.384	2.556
	L	– 0.27	1.824	0.356	0.368	0.645
T-PREC	R	– 0.15	0.316	0.438	0.453	1.070
	L	– 0.23	0.874	0.432	0.445	0.934
T-POSTC	R	0.17	0.367	0.434	0.430	0.327
	L	0.13	0.261	0.419	0.417	0.316
T-PAR	R	– 0.05	0.169	0.418	0.419	0.342
	L	– 0.06	0.169	0.406	0.411	0.364
T-OCC	R	– 0.25	1.077	0.349	0.355	0.344
	L	– 0.34	6.228	0.348	0.348	0.311
Cognitive test						
DSF		0.17	0.403	10.5 (2.7)	9.1 (2.8)	0.845
DSB		0.01	0.153	8.9 (2.4)	7.8 (1.9)	0.727
RAVLT total		– 0.33	5.870	50.1 (9.4)	61.2 (8.1)	70.547
RAVLT A7		– 0.27	6.772	10.4 (2.9)	14.0 (1.9)	144.501
SDMT		– 0.27	1.612	63.8 (12.9)	76.3 (11.2)	7.450
TMT Ratio		– 0.24	0.923	2.7 (0.9)	2.8 (1.0)	2.300
Stroop interference		0.40	35.660	1.9 (0.6)	1.5 (0.3)	0.322

Higher Stroop interference and TMT ratio scores indicate poorer performance. Means of cognitive test performance are presented as raw means rather that *z* scores, which were used in main analyses

### Whole brain fixel-based analysis

Adjusting for age and sex, whole-brain voxel-based CFE analysis indicated no statistically significant differences (*p*_*fwe*_ > 0.05) in FD, (log)FC, or FDC between mTBI and TC groups.

### Thalamocortical tract-FD

**Fig. 3:**
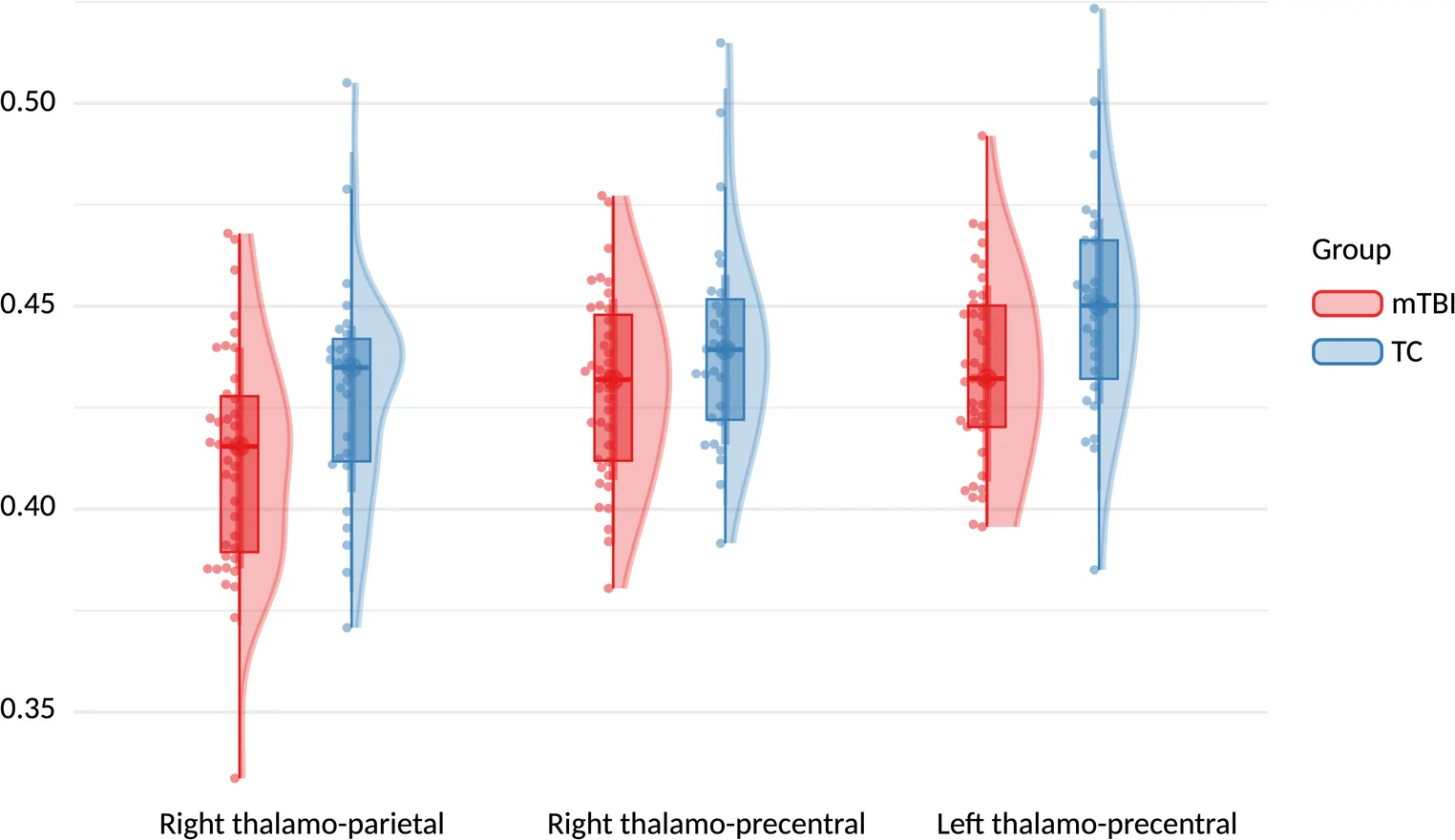
Raw distributions of mean tract-FD values for which there was evidence of a group difference, separated by group

Tract-based Bayesian analysis controlling for age and sex showed that there was moderate evidence of decreased FD of the right thalamo-precentral tract in the mTBI group compared to controls (BF_10_ = 6.849), and right thalamo-parietal tract in the mTBI group compared to controls (BF_10_ = 3.708). There was anecdotal evidence of decreased FD of left thalamo-precentral tract in the mTBI group compared to controls (BF_10_ = 1.276). There was support for the null hypothesis of no group difference in tract-FD (BF_10_ < 1/3) in 5 out of 12 tracts and inconclusive evidence for the null in 4 out of 12 tracts (1/3 < BF_10_ > 1). Figure 3 presents the raw distributions for tracts for which there was evidence of a group difference in tract FD.

### Thalamocortical tract fibre density and performance on cognitive measures

Table 3 presents an overview of the models examining tract-FD and performance on cognitive measures in each group separately, along with evidence for an interaction term between group and tract-FD. After controlling for age and sex, results from the planned Bayesian linear models revealed weak to strong evidence that the groups differed with respect to the relationships between thalamocortical tract-FD and measures of cognition. Specifically, there was consistent evidence for an interaction between group and tract-FD on measures of processing speed and attention. There was also an isolated finding of weak evidence for an interaction between T-POSTC and group when predicting performance on the TMT.

To further characterise the associations between tract-FD and cognitive performance, independent analyses were conducted within each group separately, the detailed results of which can be seen in the supplementary material. Among the models showing a significant between-group interaction, the mTBI and TC groups exhibited different patterns of association between tract-FD and performance on SDMT, DSB and DSF.

**Table 3:** Strength and direction of relationship between thalamocortical FD and performance on cognitive tests

Cognitive domain		Processing speed			Attention						Learning and Recall					Executive function					
		SDMT			DSF			DSB			RAVLT total			RAVLT A7		TMT Ratio			Stroop		
Tract	Hem	mTBI	TC	Int	mTBI	TC	Int	mTBI	TC	Int	mTBI	TC	Int	mTBI	TC Int	mTBI	TC	Int	mTBI TC Int		
T-PREF	R	–		X		–	X		–	X	+										
	L	–*		X					–	X	+										
T-PREM	R	–		X					–	X*	+*										
	L	–**	+	X**																	
T-PREC	R					–	X		–	X*	+										
	L								–												
T-POSTC	R					–			–				X					X			
	L																				
T-PAR	R								–												
	L																				
T-OCC	R																				
	L																				

Hem, hemisphere; Int, interaction, ‘-‘ indicates a negative association between tract-FD and test performance, ‘+’ indicates positive. ‘X’ indicates evidence of an interaction effect between group status and tract-FD. A sign with no * indicates an associated BF_10_ of 1–3, * = BF_10_ >3, ** = BF_10_ > 10

Bolded tracts show associations between tract-FD and cognitive performance within the mTBI group

**Fig. 4:**
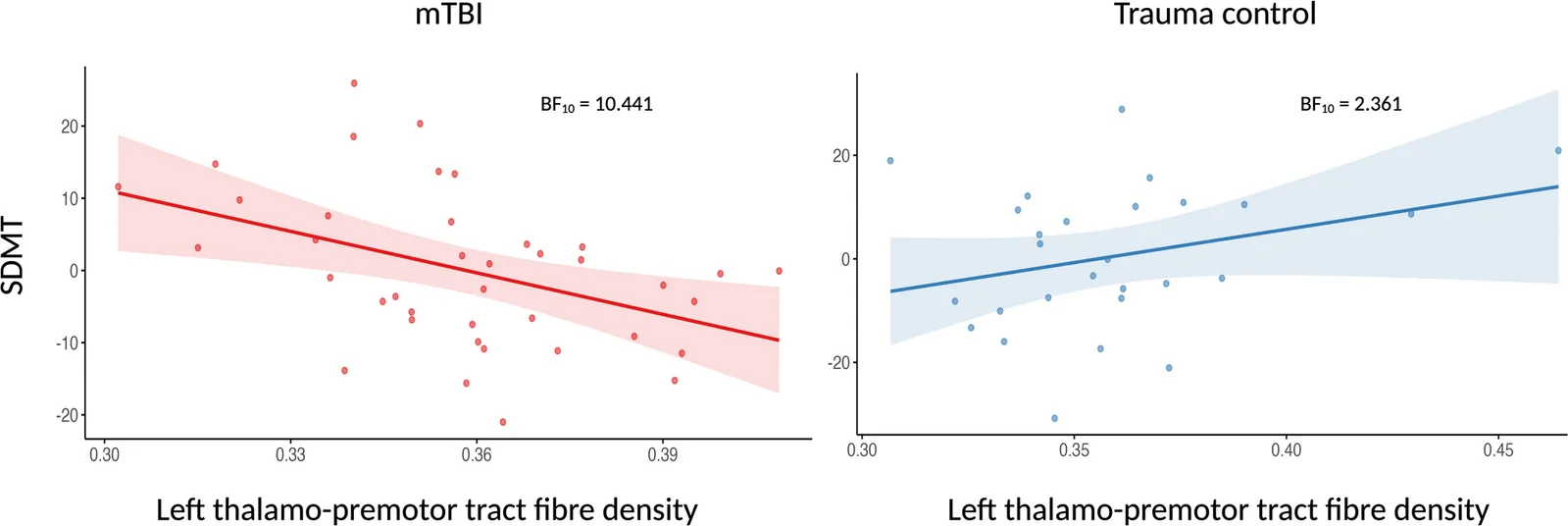
Associations between left-PREM tract-FD and performance on the SDMT, corrected for age and sex

With respect to processing speed, within the mTBI group, there was strong evidence that increased left-PREM tract-FD was associated with poorer performance on the SDMT (BF_10_ = 10.441), moderate evidence for the left T-PREF (BF_10_ = 3.005) and anecdotal evidence for both the right T-PREM (BF_10_ = 1.138), and right T-PREF (BF_10_ = 1.389). In contrast, for the TC group, there was anecdotal evidence that increased tract-FD of the left T-PREM was associated with better performance on the SDMT (BF_10_ = 2.361). The models of left-PREM FD and SDMT are presented in Fig. 4. There were no other statistically meaningful associations between tract-FD and processing speed within the TC group.

Regarding performance on the measures of attention, a different pattern emerged. For models associated with an interaction term, within the TC group, there was anecdotal level that increased tract-FD was associated with poorer performance on DSF for right T-PREC (BF_10_ = 2.783) and right T-PREF (BF_10_ = 1.743) and poorer performance on DSB for the right T-PREM (BF_10_ = 1.305), right T-PREF (BF_10_ = 1.989) and right T-PREC (BF_10_ = 2.137). There were no associations between tract-FD and performance on the DSF or DSB within the mTBI group.

As presented in Table 2, there were also several models showing independent associations between tract-FD and cognitive performance which were not associated with evidence of interaction effect. Namely, there was some evidence that increased tract-FD was associated with better verbal learning in the mTBI group across several tracts, but there were no associations within the TC group. The remaining interaction effects were not associated with independent associations between tract-FD and cognitive performance.

## Discussion

This study reports a systematic application of FBA to characterise thalamocortical white matter microstructure and associations with cognitive performance in the subacute period following an mTBI. FBA metrics were calculated at the whole-brain level, each participant’s thalamocortical tracts were delineated bilaterally, and tract-averageFD was calculated. Tract of interest analyses identified reduced FD in the bilateral thalamo-precentral and right thalamo-parietal tracts in the mTBI group compared with the TC group, despite no differences in FBA metrics at the level of the whole brain. Additionally, there was some evidence that the associations between thalamocortical tract-FD and cognitive performance differed between the two groups, despite commensurate cognitive performance between the two groups. Together, these findings suggest that subtle thalamocortical white matter changes exist in the subacute period following injury, and that aspects of the relationship between white matter microstructure and cognition may be altered at this stage of recovery.

### Decreased fibre density of thalamocortical tracts as a consequence of mTBI

The primary finding of this study was decreased FD in the bilateral thalamo-precentral tracts and the right thalamo-parietal tracts in the mTBI group compared with the TC group. The FD metric can be interpreted as approximately reflecting the volume of the intracellular component of axons oriented in a given direction, and therefore, reductions in FD may reflect a loss of axons in the mTBI group (Dhollander et al. 2021). The tracts with reduced FD predominantly connect with primary motor, premotor and parietal cortical regions. There is no clear a priori reason to expect selective vulnerability of these tracts based on injury mechanism. Rather, these findings indicate these pathways may be among those in which subtle post-injury microstructural alterations are evident at this time point. This adds to the growing body of literature reporting thalamic vulnerability in mTBI, and extends previous DTI meta-analytic evidence showing that the greatest white matter abnormalities following mTBI occur in thalamic tracts (Aoki and Inokuchi 2016).

The direction of microstructural change following mTBI is likely to depend on postinjury time course. As has been suggested in prior literature, in the acute stage following mTBI, cytotoxic oedema may increase apparent FD, whereas at later stages these processes may transition to axonal degeneration (Mito et al. 2022). The findings of our study are consistent with this proposed evolution of postinjury microstructural change and suggest that by approximately two-months post-injury, white matter pathophysiology may have progressed to subtle axonal degeneration. This interpretation further suggests that the decreases in FD previously reported at later postinjury time points (approximately 10-months) may arise earlier in the recovery trajectory, and that the subacute period may represent a transitional phase between acute and more chronic microstructural processes (Burnett et al. 2025).

Notably, no significant group differences were identified in the whole-brain fixel-wise analysis. This indicates the microstructural changes observed at this stage of recovery may be spatially distributed within specific tracts, rather than global. Tract-averaged metrics may capture subtle but spatially distributed changes across a tract, whereas whole-brain fixel-wise analyses require spatially focal effects that can survive correction for multiple comparisons across the entire white matter. Given mTBI is conceptualised as a diffuse injury, it is plausible that effects are only detectable at this time point if examined in extended white matter tracts rather than isolated fixels (Bigler et al. 2016). As such, subtle decreases in FD that occur along a tract may not manifest as significant fixel-wise differences at the whole-brain level. Consistent with this interpretation, while one study undertaking a priori tract of interest analysis reported decreases in tract-FD in the chronic postinjury time point, another reported no differences in fixel metrics at a similar time point using whole-brain analysis (Burnett et al. 2025; Wallace et al. 2020). Taken together, these findings collectively suggest that tract of interest analyses may be more sensitive to detect subtle differences in fixel metrics beyond the acute period following mTBI.

### Altered relationships between fibre density and cognition despite cognitive recovery

After identifying reduced FD within the bilateral thalamo-precentral and right thalamo-parietal tracts, linear models were used to examine whether variation in tract-FD was directly associated with cognitive performance. These models did not show consistent associations between tract-FD and cognition in either group. Specifically, for the mTBI group, reduced FD did not consistently predict worse cognitive performance. Importantly, as has been previously reported for this cohort, there were also no overall group differences in cognitive performance, which is consistent with the expectation of objective cognitive recovery at a group-level by approximately three-months following mTBI (Baird et al. 2026; Karr et al. 2014). Taken together, this suggests that normal behavioural performance can occur despite measurable microstructural changes at this time point following injury. Additional covariate analyses also demonstrated that age and sex showed expected associations with both cognitive performance and tract-FD. These findings are consistent with the broader literature and support the validity of the cognitive and FBA metrics in the present cohort, despite the absence of between-group differences in cognitive performance (Choy et al. 2020; McCarrey et al. 2016; Tinney et al. 2024). Against this background, one possible interpretation of the current findings is that at this post-acute stage of recovery, the ongoing effect of mTBI is not simply changes in white matter microstructure, but a change in how structure relates to cognitive function. To assess this possibility, associations between tract-FD and cognition were examined between the groups for all tracts, irrespective of whether they were associated with reduced FD in the mTBI group.

Models with interaction terms provided preliminary evidence that the associations between tract-FD and cognitive performance differed between groups. That is, the groups were statistically different with respect to structure-cognition relationships. Specifically, interaction effects were evident for measures of processing speed and attention (SDMT, DSF, DSB). It is notable that these were the cognitive domains most implicated in altered structure-cognition relationships in the post-acute period, given that dysfunction in processing speed and attention is the most consistently reported and enduring objective cognitive consequence of mTBI (Frencham et al. 2005). These interaction effects also involved tracts projecting to frontal, premotor, and sensorimotor cortices, which are functionally relevant for motor preparation, sensorimotor integration, and top-down attentional control (Goldberg 2022; Halassa and Kastner, 2019) Therefore, altered structure-cognition coupling in these tracts may have particular relevance for tasks requiring rapid cognitive motor responding (i.e., the SDMT) and goal-directed attention (i.e., DSF and DSB). While there is little reported data comparing associations between white matter microstructure and cognitive performance in mTBI and control groups, a pattern of altered relationships between white matter microstructure and cognition following mTBI, relative to control groups, has previously been reported (Anderson et al. 2023; Wu et al. 2018). The present results extend these findings by demonstrating that sustaining an mTBI may result in in altered brain-behaviour relationships in thalamocortical tracts, which exists even when behavioural performance has returned to ‘normal’ levels. These altered relationships appear to be most prevalent for performance on measures of processing speed and attention. However, these findings should be considered exploratory and hypothesis-generating, andeplication in larger independent samples will be important to establish the robustness and generalisability of these relationships.

Examination of these relationships within each group further illustrated heterogeneity in the direction and nature of these associations. Specifically, within the mTBI group, higher FD of bilateral frontal thalamocortical tracts (i.e., thalamo-prefrontal and thalamo-premotor) was associated with slower processing speed, whereas in the TC group, higher FD of the left thalamo-premotor tract was associated with faster processing speed. This suggests that despite commensurate levels of processing speed between the two groups, the association between tract-FD and processing speed is altered at this time point following mTBI, compared to a well-matched control group that have suffered equivalent traumatic injury without head strike. That is, normal cognitive output may be occurring in the context of altered structure-cognition associations underpinning cognition. We also observed a dissociation between tract-FD and cognition between the groups with respect to the pattern of performances on the measures of basic and high-level attention. Within the TC group, higher FD was associated with reduced basic and high-level attentional function across several tracts, whereas there were no associations within the mTBI group. While the nature of change in the relationship between tract-FD and cognition is different to that observed for the SDMT, these findings also show a dissociation between the groups with respect to the structure-cognition relationship. Together, these findings suggest that the subacute period following mTBI is characterised by normal cognitive performance at a group level alongside altered brain-cognition relationships. Rather than being associated with cognitive impairment, these altered patterns could reflect processes in which normal cognition is occurring in the post-acute period because of compensatory mechanisms rather than a return to pre-injury structural-functional relationships. Given the lack of clear mechanistic account for these findings, it is vital that further research examines the associations between structure and cognition following mTBI, as it potentially has implications for the clinical management of individuals after mTBI.

### Expected associations between FD and cognition not observed in control group

Prior work in healthy adults has reported positive relationships between FD and performance on cognitive testing across multiple domains (Tinney et al. 2024). While one positive association consistent with this literature was observed in the TC group, the overall pattern of findings was not consistently replicated, as most tract-cognition relationships were absent or negative. It is worth highlighting that there are several factors likely to be present in the TC group, who are also recovering from systemic injury as a result of their accident, which would not be expected to systematically occur in healthy adults, such as pain, fatigue, and psychological distress (Anderson and Jordan 2025; Wilde et al. 2019). Given the well-established associations between these symptoms and cognition, it is possible these factors might skew the expected ‘typical’ associations between tract-FD and cognition (Johansson et al. 2009; Massey et al. 2015). Alternatively, normative associations between white matter microstructure of thalamic tracts and cognition using the FBA method may be more heterogenous than expected, given the paucity of work examining these associations using this relatively new methodology. Due to the well-matched nature of this study’s samples, however, the reported group differences can be considered mTBI-related, rather than injury recovery-related.

### Limitations and future directions

The primary limitation of the present study was the relatively modest sample size. This may have led to limited power to detect small effects, particularly for interaction terms. Bayesian methods were applied in an attempt to overcome this limitation, as this statistical framework is more robust to small sample sizes (Wagenmakers et al. 2018). Additionally, given the cross-sectional nature of the study design, inferences about the temporal evolutions of the observed changes are not possible. Therefore, longitudinal studies are required to clarify whether these changes are transient or reflect more stable changes.

Furthermore, a notable limitation of the tract-of-interest analyses is that only FD could be examined. FC is defined in template space from the registration deformation field and therefore could not be quantified within the subject-space tracts that were derived using TractSeg. Future studies employing template-space tract definitions may be able to examine complementary microstructural (FD) and macrostructural (FC) changes within thalamocortical pathways.

## Conclusions

This study has demonstrated that at approximately two-months following injury, mTBI is associated with changes in the microstructural features of specific thalamocortical white matter tracts, which can be interpreted as evidence of axonal pathology. This novel finding extends previous literature reporting thalamic changes following injury by suggesting that subtle post-injury pathophysiology is evident in a period toward the end of the expected cognitive recovery period. There was also evidence of altered associations between thalamocortical white matter and cognition following mTBI in the post-acute period, despite cognition being ‘normal’ at a group level. The majority of these dissociations occurred for performance on measures of processing speed and attention, which previous studies indicate are the last cognitive domains to recover following injury. The mechanism and consequences of these changes are not clear and warrant future investigation.

## Supplementary Information

Below is the link to the electronic supplementary material.

Supplementary Material 1

## Data Availability

The datasets generated during and/or analysed for the current study are not publicly available due to patient privacy and possible re-identification but are available from the corresponding author on reasonable request.
