# Supplementary material for: Thalamocortical white matter microstructure and cognition following mild traumatic brain injury: A fixel-based analysis
Source: Brain Struct Funct. 2026 Sep 26;231(8):142. doi: 10.1007/s00429-026-03193-7 (PMC13615928; doi:10.1007/s00429-026-03193-7)
Supplement: Supplementary file 1 [file 429_2026_3193_MOESM1_ESM.docx]

**Thalamocortical White Matter Microstructure and Cognition Following Mild Traumatic Brain Injury: A Fixel-Based Analysis**

*Supplementary Material*

**Maggie Baird^1,2^, , Marc L Seal, PhD^2,6^, Richard Beare, PhD^2,3^, Joseph Yuan-Mou Yang, PhD^4-6 *^Jacqueline F. I. Anderson, PhD^1,7 *^**

* *Joint senior author*

**Affiliations**

^1^Melbourne School of Psychological Sciences, The University of Melbourne, Victoria, Australia

^2^Developmental Imaging, Clinical Sciences, Murdoch Children’s Research Institute, Parkville, Melbourne, Australia

^3^National Centre for Healthy Ageing and Peninsula Clinical School, Monash University, Melbourne, Australia

^4^Department of Neurosurgery, Neuroscience Advanced Clinical Imaging Service (NACIS), The Royal Children’s Hospital, Melbourne, Australia

^5^Neuroscience Research, Murdoch Children’s Research Institute, Parkville, Melbourne, Australia

^6^Department of Pediatrics, The University of Melbourne, Parkville, Melbourne, Australia

^7^Psychology Department, The Alfred hospital, Prahran, Victoria, Australia

**Corresponding Author:**

A/Prof Jacqueline Anderson Melbourne School of Psychological Sciences, The University of Melbourne, Redmond Barry Building, Parkville, Victoria, 3052, email:

| **Supplementary Table.** Bayesian correlation between thalamocortical tract FD and time since injury | | | |
| --- | --- | --- | --- |
| Subregion | Hem | *r* | BF_10_ |
| T-PREF | R | 0.05 | 0.165 |
|  | L | -0.03 | 0.158 |
| T-PREM | R | 0.03 | 0.158 |
|  | L | -0.02 | 0.55 |
| T-PREC | R | 0.05 | 0.166 |
|  | L | 0.06 | 0.169 |
| T-POSTC | R | 0.13 | 0.257 |
|  | L | -0.03 | 0.157 |
| T-PAR | R | 0.06 | 0.172 |
|  | L | -0.02 | 0.155 |
| T-OCC | R | -0.05 | 0.167 |
|  | L | -0.11 | 0.268 |

| **Supplementary Table.** Bayesian correlation between time since injury and cognitive performance | | |
| --- | --- | --- |
| Cognitive test | *r* | BF_10_ |
| DSF | -0.02 | 0.155 |
| DSB | 0.03 | 0.157 |
| RAVLT total | 0.11 | 0.230 |
| RAVLT A7 | 0.09 | 0.195 |
| SDMT | 0.05 | 0.163 |
| TMT Ratio | -0.01 | 0.153 |
| Stroop interference | 0.03 | 0.158 |
| *Note.* A higher Stroop interference score indicates *poorer* performance. | | |

| **Supplementary Table.** Relative evidence of interaction effect between group and tract-FD when prediciting cognitive performance | | | | | | | | | | | | | | | |  |
| --- | --- | --- | --- | --- | --- | --- | --- | --- | --- | --- | --- | --- | --- | --- | --- | --- |
|  | Cognitive test |  | DSF | | DSB | | RAVLT total | | RAVLT A7 | | SDMT | | TMT Ratio | | Stroop | |
|  | Tract |  | Mean [95% CI] | BF_10_ | Mean [95% CI] | BF_10_ | Mean [95% CI] | BF_10_ | Mean [95% CI] | BF_10_ | Mean [95% CI] | BF_10_ | Mean [95% CI] | BF_10_ | Mean [95% CI] | BF_10_ |
|  | T-PREF | R | -6.37 [-15.06, 1.82] | 1.487 | -8.93 [-17.83, 0.52] | 2.321 | -2.17 [-9.74, 5.23] | 0.475 | -0.03 [-7.36, 7.34] | 0.425l | 3.79 [-1.91, 10.56] | 1.130 | -1.47 [-9.89, 6.57] | 0.410 | 0.35 [-7.38, 8.07] | 0.366 |
|  |  | L | -4.51 [-12.56, 3.14] | 0.853 | -7.18 [-15.45, 0.64] | 2.494 | 1.00 [-6.13, 8.02] | 0.385 | 1.67 [-5.18, 8.63] | 0.478 | 5.46 [-1.81, 13.02] | 1.309 | -1.35 [-9.15, 6.40] | 0.437 | -0.61 [-7.63, 6.54] | 0.371 |
|  | T-PREM | R | -4.40 [-11.29, 2.89] | 0.987 | -6.72 [-13.84, -0.04] | 3.117 | -2.75 [-8.90, 3.23] | 0.573 | -1.21 [-7.28, 4.63] | 0.456 | 4.47 [-0.90, 12..00] | 2.436 | -0.78 [-7.57, 5.93] | 0.414 | 0.96 [-5.42, 7.32] | 0.382 |
|  |  | L | -3.75 [-11.93, 4.23] | 0.675 | -3.36 [-11.50, 4.26] | 0.618 | 0.75 [-6.56, 6.49] | 0.390 | 1.96 [-5.10, 9.17] | 0.456 | 10.71 [2.89, 18.85] | 21.84 | -2.49 [-10.40, 5.34] | 0.474 | -0.31 [-7.96, 7.05] | 0.360 |
|  | T-PREC | R | -6.22 [-15.21, 2.26] | 1.165 | -10.49 [-19.81, -1.83] | 8.508 | -4.67 [-12.91, 3.03] | 0.837 | -1.49 [-9.36, 6.05] | 0.453 | -1.20 [-9.66, 6.82] | 0.446 | 4.45 [-3.90, 13.11] | 0.676 | 0.59 [-7.46, 8.53] | 0.364 |
|  |  | L | -3.78 [-12.87, 4.91] | 0.596 | -7.67 [-16.95, 1.06] | 2.065 | -2.97 [-11.34, 5.10] | 0.542 | 0.67 [-7.13, 8.51] | 0.435 | -0.77 [-9.14, 7.66] | 0.392 | 3.31 [-5.51. 12.26] | 0.499 | 0.12 [-8.37, 8.56] | 0.354 |
|  | T-POSTC | R | -1.57 [-7.02, 3.79] | 0.453 | -4.59 [-10.51, 0.91] | 1.725 | -3.82 [-9.01, 1.30] | 1.293 | -0.66 [-5.61, 4.13] | 0.435 | -1.81 [-7.28, 3.35] | 0.525 | 5.49 [-0.15, 11.23] | 2.831 | -0.87 [-6.12, 4.28] | 0.371 |
|  |  | L | 0.99 [-5.82, 7.67] | 0.425 | -3.11 [-10.11, 3.58] | 0.698 | -3.22 [-9.45, 2.75] | 0.712 | 0.33 [-5.71, 6.25] | 0.435 | -3.88 [-10.36, 2.27] | 0.879 | 3.59 [-3.06, 10.33] | 0.701 | -1.83 [-8.18, 4.34] | 0.418 |
|  | T-PAR | R | -2.56 [-10.64, 5.22] | 0.521 | -6.31 [-14.64, 1.54] | 1.630 | -4.23 [-11.61, 2.78] | 0.841 | 0.09 [-6.83, 7.01] | 0.421 | -2.13 [-9.63, 5.27] | 0.467 | 4.63 [-3.25, 12.69] | 0.773 | -1.11 [-8.43, 6.33] | 0.377 |
|  |  | L | 0.71 [-7.33, 8.55] | 0.420 | -2.95 [-11.33, 4.82] | 0.561 | -2.28 [-9.71, 4.78] | 0.477 | 1.38 [-5.65, 8.42] | 0.466 | -3.41 [-11.28, 4.08] | 0.634 | 3.39 [-4.62, 11.55] | 0.557 | -1.08 [-8.71, 6.30] | 0.355 |
|  | T-OCC | R | 1.13 [-5.83, 8.13] | 0.460 | -2.64 [-9.70, 4.09] | 0.581 | -2.06 [-8.46, 4.24] | 0.495 | 1.03 [-5.29, 7.22] | 0.430 | 1.86 [-4.58, 8.27] | 0.409 | 0.65 [-6.37, 4.30] | 0.375 | 0.24 [-6.32, 6.91] | 0.366 |
|  |  | L | 2.91 [-3.37, 9.29] | 0.654 | -1.32 [-7.71, 4.84] | 0.460 | 1.12 [-4.45, 6.94] | 0.432 | 1.45 [-4.08, 7.08] | 0.478 | 1.50 [-4.41, 7.47] | 0.482 | -1.32 [-7.64, 4.82] | 0.413 | 1.50 [-4.45, 7.32] | 0.417 |

| **Supplementary Table.** Relative evidence for relationships between thalamocortical tract fibre density and DSF performance | | | | | |
| --- | --- | --- | --- | --- | --- |
|  |  | mTBI |  | TC |  |
| Subregion | Hem | Posterior Mean [95%CI] | BF_10_ | Posterior Mean [95%CI] | BF_10_ |
| T-PREF | R | 4.23 [-8.32, 17.16] | 0.563 | -9.53 [-23.21, 2.94] | 1.743 |
|  | L | 2.95 [-7.74, 13.98] | 0.532 | -6.34 [-20.17, 6.22] | 0.810 |
| T-PREM | R | 3.86 [-6.14, 14.29] | 0.610 | -4.27 [-14.15, 4.86] | 0.741 |
|  | L | 1.50 [-10.27, 13.64] | 0.462 | -5.74 [-17.63, 5.22] | 0.886 |
| T-PREC | R | -1.28 [-13.92, 11.22] | 0.471 | -11.17 [-24.54, 0.91] | 2.783 |
|  | L | -1.51 [-14.41, 11.08] | 0.461 | -6.51 [-20.52, 6.10] | 0.849 |
| T-POSTC | R | -3.69 [-11.45, 3.38] | 0.708 | -6.31 [-15.43, 1.99] | 1.565 |
|  | L | -4.92 [-13.57, 3.35] | 0.925 | -1.83 [-12.80, 8.45] | 0.525 |
| T-PAR | R | -3.02 [-12.81, 6.30] | 0.506 | -6.46 [-20.05, 5.47] | 0.889 |
|  | L | -5.46 [-15.83, 4.38] | 0.781 | -2.39 [-15.06, 9.48] | 0.528 |
| T-OCC | R | 1.09 [-6.60, 8.81] | 0.444 | 2.62 [-8.91, 14.66] | 0.543 |
|  | L | -1.86 [-10.27, 6.35] | 0.459 | 3.38 [-6.30, 13.60] | 0.632 |
| *Note.* mTBI, mild traumatic brain injury; TC, trauma control; 95% CI, 95% credible interval; DSF, digit span forward. **Bolded** models indicate BF_10_ > 3. | | | | | |

| **Supplementary Table.** Relative evidence for relationships between thalamocortical tract fibre density and DSB performance | | | | | |
| --- | --- | --- | --- | --- | --- |
|  |  | mTBI |  | TC |  |
| Subregion | Hem | Posterior Mean [95%CI] | BF_10_ | Posterior Mean [95%CI] | BF_10_ |
| T-PREF | R | 4.22 [-8.37, 17.30] | 0.556 | -9.06 [-21.18, 2.12] | 1.989 |
|  | L | 2.94 [-7.89, 14.16] | 0.514 | -9.94 [-22.47, 1.49] | 2.248 |
| T-PREM | R | 3.96 [-6.17, 14.72] | 0.588 | 5.71 [-14.43, 2.38] | 1.305 |
|  | L | 1.50 [-10.46, 13.38] | 0.437 | -4.21 [-14.69, 5.48] | 0.724 |
| T-PREC | R | -1.14 [-13.81, 11.38] | 0.445 | -9.09 [-20.90, 1.71] | 2.137 |
|  | L | -1.57 [-14.60, 11.15] | 0.429 | -7.24 [-19.64, 4.13] | 1.222 |
| T-POSTC | R | -3.69 [-11.48, 3.57] | 0.692 | -5.11 [-13.11, 2.19] | 1.366 |
|  | L | -4.99 [-13.43, 3.11] | 0.906 | -4.61 [-14.78, 4.72] | 0.838 |
| T-PAR | R | -2.91 [-12.88, 6.49] | 0.507 | -8.45 [-20.62, 2.52] | 1.749 |
|  | L | -5.43 [-15.61, 4.07] | 0.805 | -3.50 [-14.98, 7.27] | 0.625 |
| T-OCC | R | 1.19 [-6.50, 9.04] | 0.423 | -5.55 [-16.67, 4.66] | 0.903 |
|  | L | -1.84 [-10.24, 6.23] | 0.462 | -4.55 [-13.92, 3.88] | 0.895 |
| *Note.* mTBI, mild traumatic brain injury; TC, trauma control; 95% CI, 95% credible interval; DSB, digit span backward. **Bolded** models indicate BF_10_ > 3. | | | | | |

| **Supplementary Table.** Relative evidence for relationships between thalamocortical tract fibre density and RAVLT total performance | | | | | |
| --- | --- | --- | --- | --- | --- |
|  |  | mTBI |  | TC |  |
| Subregion | Hem | Posterior Mean [95%CI] | BF_10_ | Posterior Mean [95%CI] | BF_10_ |
| T-PREF | R | 7.59 [-3.73, 19.40] | 1.218 | -4.31 [-16.43, 7.33] | 0.474 |
|  | L | 5.86 [-3.75, 16.31] | 1.003 | 1.11 [-11.48, 13.79] | 0.363 |
| T-PREM | R | 9.81 [0.53, 19.40] | 4.546 | -0.11 [-9.31, 8.92] | 0.367 |
|  | L | 5.60 [-4.71, 16.53] | 0.808 | 3.40 [-7.17, 13.98] | 0.446 |
| T-PREC | R | 7.16 [-3.71, 18.96] | 1.055 | -4.27 [-15.98, 6.96] | 0.474 |
|  | L | 5.94 [-5.11, 17.58] | 0.786 | -2.37 [-14.89, 9.74] | 0.408 |
| T-POSTC | R | 1.92 [-4.61, 8.67] | 0.523 | -3.53 [-11.76, 4.42] | 0.535 |
|  | L | 1.69 [-5.23, 8.85] | 0.488 | -2.33 [-12.76, 7.98] | 0.404 |
| T-PAR | R | 3.81 [-4.54, 12.69] | 0.653 | -5.02 [-17.53, 6.80] | 0.533 |
|  | L | 3.56 [-5.18, 12.58] | 0.646 | 0.20 [-12.06, 12.25] | 0.356 |
| T-OCC | R | 2.99 [-3.65, 10.11] | 0.651 | -4.02 [-15.65, 7.36] | 0.466 |
|  | L | 1.82 [-5.42, 9.07] | 0.501 | 0.84 [-8.76, 10.56] | 0.356 |
| *Note.* mTBI, mild traumatic brain injury; TC, trauma control; 95% CI, 95% credible interval; RAVLT, Rey Auditory Verbal Learning Test. **Bolded** models indicate BF_10_ > 3. | | | | | |

| **Supplementary Table .** Relative evidence for relationships between thalamocortical tract fibre density and RAVLT A7 performance | | | | | |
| --- | --- | --- | --- | --- | --- |
|  |  | mTBI |  | TC |  |
| Subregion | Hem | Posterior Mean [95%CI] | BF_10_ | Posterior Mean [95%CI] | BF_10_ |
| T-PREF | R | 3.05 [-8.30, 14.96] | 0.522 | 1.11 [-10.12, 12.54] | 0.481 |
|  | L | 1.03 [-9.01, 11.10] | 0.468 | 2.73 [-8.92, 14.77] | 0.537 |
| T-PREM | R | 4.82 [-4.29, 14.42] | 0.817 | 0.896 [-7.62, 9.60] | 0.486 |
|  | L | 2.07 [-8.59, 12.97] | 0.467 | 4.83 [-5.25, 15.50] | 0.791 |
| T-PREC | R | 3.51 [-7.47, 15.01] | 0.544 | -0.42 [-11.37, 10.54] | 0.499 |
|  | L | 1.95 [-9.37, 13.52] | 0.458 | 2.20 [-9.67, 14.18] | 0.542 |
| T-POSTC | R | -0.26 [-6.88, 6.35] | 0.430 | -1.15 [-8.93, 6.48] | 0.498 |
|  | L | 0.48 [-6.89, 7.61] | 0.418 | 1.58 [-8.16, 11.48] | 0.505 |
| T-PAR | R | 1.76 [-6.77, 10.59] | 0.485 | 1.89 [-9.33, 13.52] | 0.507 |
|  | L | 0.59 [-8.22, 9.38] | 0.442 | 3.19 [-7.90. 14.88] | 0.559 |
| T-OCC | R | 1.01 [-5.80, 7.93] | 0.447 | 2.35 [-8.61, 13.45] | 0.522 |
|  | L | -0.08 [-7.55, 7.24] | 0.429 | 2.19 [-7.06, 11.31] | 0.524 |
| *Note.* mTBI, mild traumatic brain injury; TC, trauma control; 95% CI, 95% credible interval; RAVLT, Rey Auditory Verbal Learning Test. **Bolded** models indicate BF_10_ > 3. | | | | | |

| **Supplementary Table** Relative evidence for relationships between thalamocortical tract fibre density and SDMT performance | | | | | |
| --- | --- | --- | --- | --- | --- |
|  |  | mTBI |  | TC |  |
| Subregion | Hem | Posterior Mean [95%CI] | BF_10_ | Posterior Mean [95%CI] | BF_10_ |
| T-PREF | R | -8.22 [-19.90, 2.62] | 1.389 | -2.18 [-15.73, 10.85] | 0.487 |
|  | L | -9.18 [-19.29, -0.09] | 3.005 | 4.17 [-9.44, 18.49] | 0.570 |
| T-PREM | R | -6.02 [-15.55, 2.61] | 1.138 | 3.44 [-6.65, 14.00] | 0.580 |
|  | L | -12.94 [-23.77, -2.48] | 10.441 | 8.39 [-0.43, 20.93] | 2.361 |
| T-PREC | R | -2.34 [-13.52, 8.32] | 0.488 | -3.49 [-16.68, 9.21] | 0.549 |
|  | L | -2.30 [-13.53, 8.67] | 0.481 | -2.72 [-16.69, 11.00] | 0.521 |
| T-POSTC | R | 1.26 [-5.05, 7.57] | 0.460 | -1.40 [-10.32, 7.53] | 0.497 |
|  | L | 3.73 [-3.03, 10.97] | 0.790 | -2.59 [-14.35, 8.79] | 0.529 |
| T-PAR | R | -2.28 [-10.88, 6.01] | 0.496 | -5.35 [-19.04, 7.67] | 0.658 |
|  | L | 4.05 [-4.42, 12.64] | 0.699 | -1.19 [-14.45, 12.03] | 0.489 |
| T-OCC | R | -3.28 [-11.98, 7.05] | 0.436 | -5.22 [-18.70, 7.26] | 0.530 |
|  | L | 0.50 [-6.44, 7.58] | 0.429 | 2.52 [-8.01, 13.46] | 0.525 |
| *Note.* mTBI, mild traumatic brain injury; TC, trauma control; 95% CI, 95% credible interval; SDMT, Symbol Digits Modalities Test. **Bolded** models indicate BF_10_ > 3. | | | | | |

| **Supplementary Table.** Relative evidence for relationships between thalamocortical tract fibre density and TMT Ratio | | | | | |
| --- | --- | --- | --- | --- | --- |
|  |  | mTBI |  | TC |  |
| Subregion | Hem | Posterior Mean [95%CI] | BF_10_ | Posterior Mean [95%CI] | BF_10_ |
| T-PREF | R | 5.55 [-6.55, 17.88] | 0.536 | 2.68 [-8.52, 14.53] | 0.538 |
|  | L | 3.05 [-7.47, 13.84] | 0.408 | 0.04 [-11.93, 11.97] | 0.480 |
| T-PREM | R | 1.88 [-8.07, 11.77] | 0.378 | -0.10 [-8.79, 8.38] | 0.480 |
|  | L | 2.38 [-8.98, 13.56] | 0.371 | -1.44 [-11.93, 8.37] | 0.508 |
| T-PREC | R | -0.95 [-13.10, 11.15] | 0.343 | 4.23 [-6.77, 15.77] | 0.689 |
|  | L | 0.41 [-11.83, 12.56] | 0.333 | 3.06 [-8.40, 14.94] | 0.572 |
| T-POSTC | R | -5.00 [-11.99, 1.74] | 0.943 | 3.63 [-3.94, 11.80] | 0.781 |
|  | L | -2.33 [-10.35, 5.25] | 0.396 | 1.68 [-8.06, 11.80] | 0.524 |
| T-PAR | R | -4.72 [-14.17, 4.35] | 0.568 | 1.06 [-10.23, 12.39] | 0.507 |
|  | L | -1.37 [-10.96, 7.90] | 0.337 | 1.33 [-10.10, 13.06] | 0.515 |
| T-OCC | R | -4.63 [-12.14, 2.61] | 0.768 | -0.66 [-11.77, 10.11] | 0.487 |
|  | L | 2.42 [-5.52, 10.48] | 0.413 | 2.29 [-6.78, 11.61] | 0.546 |
| *Note.* mTBI, mild traumatic brain injury; TC, trauma control; 95% CI, 95% credible interval; TMT, Trail Making Test. **Bolded** models indicate BF_10_ > 3. | | | | | |

| **Supplementary Table .** Relative evidence for relationships between thalamocortical tract fibre density and Stroop interference | | | | | |
| --- | --- | --- | --- | --- | --- |
|  |  | mTBI |  | TC |  |
| Subregion | Hem | Posterior Mean [95%CI] | BF_10_ | Posterior Mean [95%CI] | BF_10_ |
| T-PREF | R | 3.76 [-10.40, 18.56] | 0.435 | 1.67 [-6.52, 9.66] | 0.456 |
|  | L | 3.05 [-9.09, 15.74] | 0.443 | -1.66 [-10.51, 6.80] | 0.449 |
| T-PREM | R | 0.82 [-10.92, 12.46] | 0.393 | 0.86 [-5.50, 7.31] | 0.418 |
|  | L | 0.79 [-12.07, 13.77] | 0.384 | -1.22 [-8.68, 6.19] | 0.432 |
| T-PREC | R | 3.85 [-10.11, 18.10] | 0.465 | 2.67 [-5.22, 10.77] | 0.522 |
|  | L | 4.68 [-9.58, 19.35] | 0.480 | 2.12 [-6.35, 10.74] | 0.483 |
| T-POSTC | R | 3.26 [-4.77, 11.59] | 0.525 | 1.73 [-3.79, 7,47] | 0.505 |
|  | L | 4.86 [-4.03, 14.05] | 0.684 | 0.99 [-6.10, 8.15] | 0.427 |
| T-PAR | R | 3.65 [-7.10, 14.84] | 0.473 | 0.11 [-8.18, 8.32] | 0.421 |
|  | L | 6.16 [-4.87, 17.78] | 0.717 | 2.53 [-5.66, 10.92] | 0.518 |
| T-OCC | R | -0.24 [-8.83, 8.07] | 0.377 | 0.09 [-7.85, 8.04] | 0.414 |
|  | L | 0.34 [-9.06, 9.63] | 0.375 | 3.09 [-3.39, 9.83] | 0.658 |
| *Note.* mTBI, mild traumatic brain injury; TC, trauma control; 95% CI, 95% credible interval. **Bolded** models indicate BF_10_ > 3. | | | | | |
